# Chronic Pelvic Pain: A Comprehensive Review

**DOI:** 10.7759/cureus.30691

**Published:** 2022-10-26

**Authors:** Anup Juganavar, Ketav S Joshi

**Affiliations:** 1 Obstetrics and Gynaecology, Jawaharlal Nehru Medical College, Datta Meghe Institute of Medical Sciences, Wardha, IND

**Keywords:** gastroentrologic pain, pelvic pain, etiologies of cpp, pelvic inflammatory diseases, gynaecological pain

## Abstract

Chronic pelvic pain (CPP) is explained as a complaint of cyclic or non-cyclic pelvic pain lasting for at least six months with or without dysmenorrhea, dyspareunia, dysuria, and dyschezia. The etiology of symptoms can be categorized according to organ system involvement. Gynecological causes typically involve endometriosis-related pain, pelvic congestion syndrome, pelvic inflammatory disease, adenomyosis, hydrosalpinx, etc. Endometriosis-related pain is seldom non-cyclic and may present due to recurrent bleeding in endometriotic implants. Engorgement of veins leads to inadequate venous washout and presents chronic pelvic pain in pelvic congestion syndrome. The pressure effect of benign lesions of the uterus and cervix may lead to cyclic pain, as in uterine fibroids. Often presentation of diseases like hydrosalpinx may not present until it has overdistended or may at times present as acute pelvic pain if it undergoes torsion. Long-standing untreated pelvic inflammatory diseases in sexually active females is another cause of pelvic pain. The complaint of CPP is also shared due to the involvement of the gastrointestinal system in conditions like irritable bowel syndrome, inflammatory bowel diseases, long-standing abdominal hernias, colorectal cancer, etc. Alteration of the gut biome and dysregulated brain-gut associations lead to typical manifestations of chronic lower back pain and pelvic pain in irritable bowel syndrome. Colorectal tumors, when in the advanced stage, may spread to nearby tissues creating fistulas and affecting nearby nerves, causing pelvic, perineal, and sacral pain. Abdominal hernias with small bowel prolapse are always related to pelvic pain symptoms. Infections in the urinary tract like urethral syndrome, chronic prostatitis, and chronic recurrent cystitis present with CPP and voiding problems. Musculoskeletal etiologies, though varying in degrees, are responsible for isolated complaints of CPP. Examples include pelvic girdle pain, levator syndrome, coccygodynia, and pelvic floor prolapse.

## Introduction and background

Chronic pelvic pain (CPP) in women can be defined as cyclical or noncyclical pain lasting at least a six-month duration. Dysmenorrhea, dyspareunia, dysuria, and dyschezia are a few possible pain-related symptoms. Up to 24% of women globally suffer from CPP [1]. Most often, CPP is typically a condition that significantly alters a woman's daily activities. CPP can include the gastrointestinal, urinary, gynecological, oncology, musculoskeletal, and psychosocial systems [2]. When evaluating and treating patients, subspecialists frequently lack multidisciplinary training and knowledge of the various reasons required. Thus, a patient may have seen multiple doctors before seeking treatment, undergone many tests, and occasionally had surgery without experiencing much comfort. The lengthy wait for women to receive a diagnosis and appropriate care is one of the practical problems [3]. An evaluation-focused multidisciplinary overview is attempted in this piece. It analyses the most prominent causes of CPP, including its etiology, causes, and diagnosis.

## Review

Gynecologic

CPP in Endometriosis

Although endometriosis is a benign proliferative development process, it exhibits several characteristics of cancer, including the capacity to invade the healthy surrounding tissues, the ability to cause excruciating pain, and the propensity for recurrences. About 10 percent of childbearing-aged females have endometriosis, but the incidence is increasing due to the greater use of laparoscopy. Endometriosis is a chronic disease causing CPP and subfertility [4]. Several theories have been propounded to explain endometriosis, chief among these are the following: retrograde menstruation, coelomic metaplasia, embryologic rests, and lymphovascular spread [5]. Depending on what caused the discomfort, the pathophysiology may differ. For instance, endometriosis causes cyclical discomfort due to recurring bleeding in the endometriotic implants. Pelvic veins that are engorged and dilated in people with pelvic congestion syndrome result in an inadequate venous washout, which causes pain. About one-third of the patients are asymptomatic. Symptoms frequently coexist. Some of the classic symptom complexes include CPP (cyclical and noncyclical), dysmenorrhea, dyspareunia, pain on defecation and urination, fatigue and depression, sub/infertility [6], and bleeding disorders [7].

CPP in Uterine Fibroid

Uterine leiomyomas (fibroids or myomas) are some of the commonest benign uterine neoplasms arising from a uterus, commonly encountered in gynecological practice and clinically seen in women of reproductive age group. All fibroids begin in the myometrium, but the submucous type may grow more towards the endometrial cavity, or the subserous type may grow towards the serosal surface of the uterus. However, most tend to remain in the myometrium as in the interstitial type. Excessive menstrual bleeding, menstrual irregularities, and intermenstrual bleeding are symptoms, along with chronic pelvic pain and pressure-related gastrointestinal symptom such as bloating, increased frequency of urination, and bowel disturbance. Furthermore, they may interfere with reproductive processes, resulting in subfertility, early pregnancy loss, and difficulties in a later pregnancy [8].

CPP in Adenomyosis

One common ailment in the gynecology outpatient department is adenomyosis, often known as uterine endometriosis. Older women aged around 40 years are more prone to this disease. The disease often coexists with endometrial carcinoma, uterine leiomyomas, and pelvic endometriosis. Some women are asymptomatic, and others may show symptoms such as pelvic discomfort, backache, menorrhagia, dyspareunia, and progressively increasing dysmenorrhoea. Although adenomyomas rarely cause uterine hypertrophy and bulk symptoms, they can cause similar pain sensations by inflaming the myometrium [9]. Most gynecologists prefer MRI investigation to ultrasound in diagnosing the disease, as MRI shows a hypo or anechoic area in the uterine wall [10]. Histologic diagnosis is ultimate in diagnosing adenomyosis [11].

CPP in Hydrosalpinx

Hydrosalpinx is described as a collection of fluid in a fallopian tube lumen, thus causing its distension. The majority of hydrosalpinx patients will not have any symptoms. Therefore, individuals might not become aware of their problem until they are unable to conceive. Those with signs of hydrosalpinx may feel abnormal vaginal discharge and pelvic and abdominal pain, which may get worse during a menstrual cycle. Hydrosalpinx is most of the time bilateral. Generally, the wall of hydrosalpinx is translucent and thin. Some researchers believe that hydrosalpinx is mobile and can undergo torsion. Hydrosalpinx can be caused by sexually transmitted infections (STIs), endometriosis, previous fallopian tube surgery, and fallopian tube infection. The gynecologist can confirm hydrosalpinx by hysterosalpingogram (HSG) or laparoscopy [12].

CPP in Pelvic Inflammatory Disease (PID)

One of the upper genital tract infections, known as a pelvic inflammatory disease (PID), is most common in young women and those who are sexually active. Neisseria gonorrhoeae and Chlamydia trachomatis are popular causative organisms. If left untreated, the pelvic inflammatory disease might result in intraabdominal infection, infertility, ectopic pregnancies, and chronic pelvic pain [13]. Less than twenty-five years of age, new or several sexual partners, having unprotected sexual activity, having sexual activity with a partner who is experiencing symptoms, young age at first sexual act (under fifteen years old), or a previous history of any STIs or PID are some of the risk factors for PID [14]. The sudden development of the lower abdomen or pelvic pain in women who are actively engaged in sexual activity is the hallmark feature of PID. The signs and symptoms can be modest, such as mild lower bilateral abdomen ache that worsens with coitus, dyspareunia, abnormal uterine bleedings, dysmenorrhoea, increased frequency of micturition, menorrhagia, dysuria, or abnormal vaginal discharges [15].

CPP in Pelvic Congestion

Pelvic congestion, similar to a scrotal varicocele in men, is very commonly observed in women between the ages of 20 and 30. In general, premenstrual symptoms typically get worse, and the intensity of symptoms typically rises during the day. Additionally, patients may express intense dyspareunia or postcoital pain. Imaging procedures, including duplex venography, MRIs, and laparoscopies, can all show dilated vessels. Alongside capillary endothelium hypertrophy and proliferation, pathological findings encompass fibrosis of the tunica intima and media [16]. Ovarian suppression and embolic therapy potentially reduce clinical signs, with studies showing success rates for embolic therapy ranging from 24% to 100% [17].

Gastroenterologic

CPP in Irritable Bowel Syndrome (IBS)

IBS tends to be more common (8-41%) in women with CPP than in the general population [18]. Alterations in the gut microbiome, intestinal permeability, gut immune function, motility, visceral sensation, brain-gut connections, and psychosocial status are among the elements that play a vital role in the onset of IBS [19]. It tends to affect 10 to 20 percent of the population in general, and the preponderance is higher in women and individuals with underlying psychologic comorbidity or associated functional disorders. Peripheral and central sensitization may result from dysregulated brain-gut associations. Increased activity in brain areas responsible for emotional arousal and pain modulation is linked to central sensitization at the spinal cord and brain level [20]. Clinical manifestations include temporomandibular joint dysfunction, chronic IBS symptoms such as chronic low back pain, chronic pelvic pain, chronic headaches, vagus nerve inflammation, depression, and anxiety [21]. IBS is more common in women than men, and they are roughly three times as likely to seek medical assistance as men do [22]. The younger folks are more likely to report having IBS. Since menstruation will worsen IBS pain in about half of IBS patients, it can be challenging to differentiate IBS pain from other gynecological causes of chronic pelvic discomfort [23]. Progesterone and prostaglandin appear to be the key contributors, though the exact mechanisms by which menstruation affects IBS symptoms are not fully understood [24]. IBS is typically distinguished from some of the other non-gastrointestinal causes of pelvic pain by the presence of additional gastrointestinal symptoms in most patients. Presently, the accepted method for diagnosing IBS is according to the Rome II criteria [25].

CPP in Inflammatory Bowel Disease (IBD)

IBD is a chronic, idiopathic, inflammatory condition of the GI tract which encompasses two disorders: Crohn's disease (CD) and ulcerative colitis (UC) [26]. Patients suffering from IBD come to outpatient departments complaining of chronic lower abdominal pain and pelvic pain. Due to the fact that individuals experiencing associated gastroenterological manifestations are more likely to be referred to a gastroenterologist and are less likely to be included in obstetrical series, it is challenging to determine the actual incidence of inflammatory bowel diseases in individuals presenting with pelvic pain.

Diarrhea with urgency and tenesmus are common signs of ulcerative colitis. Blood and mucus are passed along with the loose stools. There could be a tightening lower abdominal discomfort, particularly in the left iliac fossa. This results from the colonic wall becoming tenser during contractions due to inflammation [27]. Asymmetric non-erosive arthropathy involving big joints affects about 10% of people with an acute flare of UC [28]. Pelvic discomfort could result from hip arthropathy. Rarely (5% of instances) does poor pouch function aggravate ileoanal pouch surgery after total colectomy, leading to pelvic pain [29].

Compared to UC, pain is considerably more likely to be a presenting complaint of CD. Additionally, patients are more likely to develop a bone disease such as pelvic osteomyelitis due to fistulizing illness [30] and osteonecrosis of the femur head [31,32], which can cause discomfort in the pelvis.

CPP in Colorectal Carcinoma

In the USA, the second most prevalent cause of cancer-related death is colorectal carcinoma. Every year approximately 1,30,000 new instances are detected in the US population, and it is projected that 57,000 of them pass away from this malignancy. Colorectal tumors can lead to various types of abdominal and pelvic pain. Advanced rectal tumors may spread to nearby tissues, such as the bladder and vagina, creating fistulae. They may also affect nearby nerves, causing pelvic, perineal, and sacral pain. An abdominal lump that can be vaguely felt during an examination of the abdomen and rectal examination can reveal rectal tumors. The stool has to be examined for the presence of heme occult. Colonoscopy is the best method for determining the presence of colorectal tumors [27].

CPP in Abdominal Hernias

Less than 2% of female groin hernias are direct inguinal hernias. Most groin hernias in women are indirect inguinal hernias, which comprise around 70% of all cases. Femoral hernias, which comprise about 30% of cases, are next in frequency. Internal hernias detected during laparoscopy were thought to be the source of discomfort in two of the 141 individuals in the study who had chronic pelvic pain (1.6%) [33]. Twenty white women with persistent pelvic pain had sciatic hernias, which were repaired by laparoscopy. Right-sided hernias occurred in 14 cases, left-sided hernias in five, and bilateral hernias in one. The ipsilateral ovary alone or its fallopian tube was present in every sciatic hernia. All 20 patients noted symptom alleviation during the follow-up visits [34]. Therefore, in patients complaining of persistent pelvic pain, hernias should be properly inspected, and all hernia abnormalities should be surgically repaired [35]. Enterocele refers to the peritoneal sac herniating between the vagina and rectum, which frequently contain the small bowel or the sigmoid colon. Enteroceles are frequently linked to pelvic pain symptoms, as is widely recognized [36].

Urologic

CPP in Painful Bladder Syndrome (PBS)

According to the definitions, the condition is "an unpleasant sensation (pain, pressure, discomfort) considered to be associated to the urine bladder, accompanied with symptoms of the lower urinary tract of more than six weeks period, in the absence of any infections or other identified causes" [37]. Interstitial cystitis (IC) or PBS is characterized by pain that might be localized to the suprapubic region and is frequently accompanied by increased urination, a repeated urge to urinate. Even though there are numerous theories on the etiology of PBS/IC, a multifactorial cause is the most widely accepted. Bladder epithelial damage occurs after infection, inflammation, pelvic surgery, childbirth, or urological instrumentation [38]. Pelvic pain with urinary frequency and urgency is the most common presentation of PBS/IC. PBS/IC is more common in females. Male patients with symptoms of PBS/IC are diagnosed with chronic abacterial prostatitis (CAP) [39]. The most common sign of IC is pelvic pain [40]. Fifteen percent of IC patients initially presented with pain without any urologic symptoms [41]. Some researchers have proposed that the causative agent of IC could be an activation of the process in the bladder by harmful compounds in the urine [42], an infectious process [42], an autoimmune occurrence [43,44], a traumatic etiology [45], an autoimmune phenomenon with a trigger such as one of the elements above [38], or a neuroinflammatory process [46].

CPP in Recurrent Cystitis

Three UTI bouts in the previous 12 months, or two incidents in the prior six months, are typically required to be diagnosed with recurrent cystitis. Recurrent UTIs are symptomatic UTIs that typically, but not always, occur after the clinical clearance of an earlier infection. Most females can recognize their boults of recurrent cystitis based on the symptoms (positive predictive value was 92% in one randomized control trial [RCT]). Diaphragm-spermicide use, history of recurrent UTI, and sexual activity have all been found to be significant, independent risk factors for cystitis [47].

Chronic Prostatitis (CP)

In 1995, the National Institutes of Health recognized the syndrome. When there are no anatomical abnormalities, urologic malignancies, or urinary tract infections, it is distinguished by CPP and voiding symptoms [48]. Whether leukocytes are present in expressed prostatic secretions or not, there are inflammatory and noninflammatory varieties. In a review of research articles on CP/CPP, there are a variety of treatment options that can be used, and a multi-modality therapeutic approach is favored, including the use of alpha-blockers in patients with severe voiding symptoms and physical therapy or antibiotics in newly diagnosed individuals who are antimicrobial naive [49].

Urethral Syndrome

Partial emptying and burning while passing urine are urethral syndrome symptoms, especially after sexual activity. Examining the urethra may reveal it to be tender. It is suspected that non-infectious, stenotic, or fibrous modifications in the urethra produce urethral syndrome. Grand multiparity, vaginal deliveries without episiotomy, and overall pelvic relaxation are attributed to urethral syndrome [50]. Coagulation and diathermy are the usual forms of treatment [51].

Musculoskeletal

Pelvic Girdle Pain

Pain in the posterior sacrum or the buttocks, of varying degrees, is how pelvic girdle pain manifests [52]. In most cases, it is related to recent pregnancies or pain that began while pregnant [53]. Around 1-16% of women experience pain beyond a year after giving birth [54]. Commonly a multimodal type of treatment is preferable in these types of patients. A program of exercises aimed at regaining pelvic stability is suggested for them. Whenever there is a pain in the sacroiliac joint, steroids are injected intra-articularly [55].

Levator syndrome

Levator syndrome is a group of complicated musculoskeletal illnesses, encompassing piriformis and puborectalis syndromes, typically caused by a muscle spasm of the pelvic floor musculature. Women are more prone to it. Generally speaking, the clinical presentation includes vague, dull pressure or aches that may get worse when you sit or lie down. It frequently relates to insufficient evacuation [56]. A diagnosis is determined by palpating the muscles and any underlying tenderness. In as many as 68% of patients, digital massage has been linked to symptom relief [57]. Additionally, case studies show improvement following a Botox injection [58].

Coccygodynia

When the coccyx is moved, it can cause coccygodynia, which is aching at or surrounding the coccyx. It frequently occurs after localized trauma, lengthy sitting, or cycling. It could get worse when you sit, bend over, or get up, as well as when you have sexual activity or while defecating. Coccygodynia may develop due to increased pelvic floor tension and limited coccyx movement. In about 30% of cases, the cause could have been unknown. After a local anesthetic injection, pain reduction can confirm a diagnosis [59]. It is highly advised to avoid coccygectomy.

Pelvic Floor Prolapse

In up to 50% of multiparous women, pelvic prolapse may be present. The multifactorial combinations of aging, trauma, devascularization, altered collagen composition, and decreased estrogen levels are thought to cause pelvic prolapse [60]. In young premenopausal individuals with pelvic floor prolapse, collagen content has been found to be lower than in normal controls [61]. Mucous discharge and incontinence can both be symptoms of rectal prolapse. Surgical management is considered the gold standard method in the treatment of prolapse cases. However, some patients with anterior pelvic floor prolapse may find relief from their symptoms by using a pessary.

Infectious causes

Pelvic pain may be caused by gynecological conditions such as chlamydia, gonorrhea, syphilis, HIV/AIDS, trichomoniasis, vaginitis, and genital herpes [62]. Objective findings could be limited, and partner-untreated reinfection is frequent. If there is no significant level of suspicion, treatment might be postponed. Persistent infections may cause infertility.

Although CPP is most often seen in women, it usually doesn't get diagnosed properly and hence is treated inadequately. Newer treatment strategies are needed to treat chronic pelvic pain disease. Given below are some of the recent studies on the treatment of CPP (Table 1).

**Table 1:** Newer strategies used in the management of chronic pelvic pain b.i.d. = two times a day

Serial Number	Objectives of the study	Type of Study	Implications	[Citation] Author, Year
1	Evaluate pain improvement in chronic pelvic pain syndrome (CPPS) after botulinum toxin type-A (BTX-A) treatment.	Systematic review and pooled meta-analysis	Pooled meta-analysis of prospective studies demonstrating a statistically significant pain relief after BTX-A injection compared to baseline values for CPPS in all evaluated cohorts	[63] Panunzio et al., 2022
2	To compare the effectiveness of two different treatment regimens of dydrogesterone in the management of endometriosis-related chronic pelvic pain (CPP).	Observational, prospective cohort study	Prolonged cyclical and continuous treatment regimens of dydrogesterone therapy both demonstrated a pronounced and similar reduction in the severity of chronic pelvic pain and dysmenorrhea and led to marked improvements in all study parameters related to the quality of life and sexual well-being	[64] Sukhikh et al., 2021
3	Evaluate the effects of paresthesia-independent 10-kHz spinal cord stimulation (SCS) in subjects with CPP.	Prospective, single-arm pilot study	Therapeutic modality of paresthesia-independent stimulation with 10-kHz SCS could potentially treat patients with CPP while improving their quality of life	[65] Tate et al., 2021
4	Investigate the effect of extracorporeal shock wave therapy (ESWT) combined with pharmacotherapy in the treatment of chronic prostatitis/chronic pelvic pain syndrome (CP/CPPS).	Randomized control trial (RCT)	ESWT in combination with pharmacotherapy could improve the treatment outcome in patients with CP/CPPS.	[66] Rayegani et al., 2020
5	To compare the clinical efficacies of inferior hypogastric plexus blockade and acupuncture in the management of idiopathic CPP.	RCT	Inferior hypogastric blockade had a 72.6% success rate and showed a significantly higher effect on reducing pain intensity in a short period of time in the management of CPP, compared to acupuncture.	[67] Amin et al., 2015
6	To determine the effect of neurogenic acupoint dry cupping therapy on high sensitive C-reactive protein (hs-CRP) level, pain perception & intensity, and life impact of pelvic pain in women with CPP, with regard to the biological and neurophysiological impacts of dry cupping on acupoint.	RCT	Neurogenic acupoint cupping therapy had significantly improving effects on the degree of inflammation, pain perception & intensity, and life impact of pelvic pain in women with CPP.	[68] Abdulaziz et al., 2021
7	To evaluate and compare the eficacy of therapeutic ultrasound (TUS) and injection of local anesthetic (IA) to improve pain in women with abdominal myofascial syndrome secondary to CPP.	RCT	TUS and IA were effective in reducing clinical pain and improving quality of life through the variables analyzed among study participants. There was no significant difference between groups	[69] Baltazar et al., 2022
8	To evaluate to use of metronidazole in treatment of acute pelvic Inflammatory disease with ceftriaxone and doxycycline	RCT	Addition of metronidazole to ceftriaxone and doxycycline was well tolerated and resulted in reduced endometrial anaerobes, decreased Mycoplasma genitalium, and reduced pelvic tenderness compared to ceftriaxone and doxycycline.	[70] Wiesenfeld et al., 2021
9	Assessing the efficacy and safety of tenapanor 50 mg b.i.d. for the treatment of patients with constipation-predominant irritable bowel syndrome (IBS-C).	Phase 3, double-blind clinical trial study	Tenapanor 50 mg b.i.d. improved IBS-C symptoms and was generally well tolerated, offering a potential new treatment option for patients with IBS-C	[71] Chey et al., 2020
10	To compare the efficacy of an on-demand alverine citrate/simeticone (ACS) treatment vs. that of usual treatments.	RCT	On-demand ACS treatment led to a greater improvement of quality of life (QoL), reduced the burden of the disease, and was more effective for IBS symptom relief than usual treatments	[72] Ducrotte et al., 2014
11	7-Year data from the PRECiSE 4 study: reinduction with certolizumab pegol in patients with Crohn’s disease experiencing disease exacerbation	PRECiSE 4 clinical trials	Certolizumab pegol was effective in many patients who previously discontinued certolizumab pegol for lack or loss of response. Thus, discontinuation of therapy may not always be necessary	[73] Lee et al., 2016
12	To determine the efficacy and safety of pelvic floor Myofascial Physical Therapy (MPT) in women with newly-symptomatic interstitial cystitis/painful bladder syndrome(IC/PBS), as compared to Global Therapeutic Massage (GTM).	RCT	A significantly higher proportion of women with IC/PBS responded to treatment with MPT than with GTM. MPT may be a beneficial therapy in women with this syndrome.	[74] FitzGerald et al., 2012
13	To evaluate the efficacy and safety of intravesical KRP-116D, 50% dimethyl sulfoxide solution compared with placebo, in IC/PBS patients.	Randomized controlled parallel-group comparative trial	KRP-116D improves symptoms, voiding parameters, and global response assessment, compared with placebo, and has a well-tolerated safety profile in IC/PBS patients with the bladder-centric phenotype.	[75] Yoshimura et al., 2021
14	To study the effect of platelet-rich plasma as an intravesical therapy to prevent the recurrence of bacterial cystitis.	RCT	Platelet-rich plasma can significantly decrease the recurrence of bacterial cystitis up to a year after instillation without any side effects.	[76] Mirzaei et al., 2019

## Conclusions

The scope of CPP in the world is daunting. One-quarter of women of reproductive age complain of CPP of greater than one year in duration, and a very large number of gynecological visits to healthcare providers are related to pelvic pain. The patient often describes the pain of pelvic diseases vaguely and inconsistently, which reflects the multiple factors having a role in its pathogenesis. Unfortunately, it is approached as a specific entity without distinguishing between the many symptoms typically associated with specific etiologies. Therefore, sound research about the clinical reflections of these varieties of causes reinforces the necessity of consolidated literature as well as thoughtful history in evaluating patients with CPP complaints. Thus, this petit review may aid in summarizing the clinical presentations as well as the etiologies of the spectrum of CPP.
